# Structure of the uracil complex of *Vaccinia virus* uracil DNA glycosylase

**DOI:** 10.1107/S1744309113030613

**Published:** 2013-11-28

**Authors:** N. Schormann, S. Banerjee, R. Ricciardi, D. Chattopadhyay

**Affiliations:** a Department of Medicine, University of Alabama at Birmingham, Birmingham, AL 35294, USA; b Northeastern Collaborative Access Team and Department of Chemistry and Chemical Biology, Cornell University, Argonne, IL 60439, USA; c Department of Microbiology, School of Dental Medicine, Abramson Cancer Center, University of Pennsylvania, Philadelphia, PA 19104, USA

**Keywords:** uracil DNA glycosidase, *Vaccinia virus*

## Abstract

Poxvirus uracil DNA glycosylases are the most diverse members of the family I uracil DNA glycosylases (UNGs). The crystal structure of the uracil complex of *Vaccinia virus* uracil DNA glycosylase (D4) was determined at 2.03 Å resolution. One uracil molecule was located in the active-site pocket in each of the 12 noncrystallographic symmetry-related D4 subunits. Although the UNGs of the poxviruses (including D4) feature significant differences in the characteristic motifs designated for uracil recognition and in the base-excision mechanism, the architecture of the active-site pocket in D4 is very similar to that in UNGs of other organisms. Overall, the interactions of the bound uracil with the active-site residues are also similar to the interactions previously observed in the structures of human and *Escherichia coli* UNG.

## Abstract 2

The crystal structure of the uracil complex of *Vaccinia virus* uracil DNA glycosylase (D4) has been determined at 2.03 Å resolution.

## 1. Introduction

Uracil-DNA glycosylases (UDGs) are ubiquitous DNA-repair enzymes that remove uracil from DNA. The presence of uracil in DNA results from the spontaneous deamination of cytosine (Cyt) or from the misincorporation of dUMP by DNA polymerase (Kosaka *et al.*, 2007 ▶; Zharkov *et al.*, 2010 ▶) and can have potentially harmful cellular consequences. Thus, UDGs have evolved as the most common form of DNA-repair enzymes in all organisms. UDGs are classified into six families based on their substrates and amino-acid sequences (Chung *et al.*, 2003 ▶; Kosaka *et al.*, 2007 ▶). Among these, the family I UDGs, also called the UNGs, are the best characterized (Chung *et al.*, 2003 ▶; Kosaka *et al.*, 2007 ▶). These enzymes show similarity in their sequences, especially in the areas involved in substrate recognition and catalysis. For example, the primary sequence of human UNG (hUNG) has 56 and 42% identity to *Escherichia coli* UNG (eUNG) and *Herpes simplex virus* UNG (hsvUNG), respectively. The architecture of the active site and the mechanism of base excision have been established in sufficient detail from biochemical, kinetic and structural studies of UNGs and their complexes with DNA (Savva *et al.*, 1995 ▶; Parikh *et al.*, 1998 ▶; Xiao *et al.*, 1999 ▶; Werner *et al.*, 2000 ▶; Leiros *et al.*, 2005 ▶; Kaushal *et al.*, 2010 ▶). These data show that upon binding uracil-containing DNA, UNG undergoes a conformational change and the uracil is flipped out of the DNA major groove into the active site, which is formed by a set of conserved residues and is highly specific for binding uracil in DNA (Kavli *et al.*, 2002 ▶; Schärer & Jiricny, 2001 ▶). Two strictly conserved residues in the active site, an aspartic acid and a histidine, participate in the acid–base catalytic mechanism for the excision of the uracil base, and a concurrent movement of their side chains has been noted as being necessary for catalysis (Slupphaug *et al.*, 1996 ▶; Parikh *et al.*, 1998 ▶; Xiao *et al.*, 1999 ▶).

The UNGs of poxviruses are among the most interesting and divergent members of the family. The identity in the primary sequences of the UNGs of various poxviruses varies over a wide range. Thus, the UNGs of *Vaccinia virus* (the prototypic poxvirus), *Variola virus* (smallpox) and *Cowpox virus* share 99% identity in their primary sequence, but the identity decreases to 66 and 54% when these sequences are compared with those of *Sheeppox virus* and *Fowlpox virus* UNGs. The *Vaccinia virus* UNG (known as D4) shows only 20% sequence identity to both hUNG and eUNG. Moreover, the conserved motifs that are implicated in DNA binding and the catalytic mechanism in various UNGs are different in D4 (Schormann *et al.*, 2007 ▶). For example, the conserved ‘catalytic water-activating loop’ (GQDPYH) is replaced by ^66^GIDPYP^71^ in D4 and the typical ‘uracil-specificity motif’ (LLLN) is substituted by ^117^IPWN^120^. In addition, the ‘DNA-intercalation loop’ (HPSPLS*XX*R, also known as the leucine-intercalation loop) is substituted by ^181^HPAARDR^187^ in D4 (Savva *et al.*, 1995 ▶; Mol *et al.*, 1995 ▶; Parikh *et al.*, 1998 ▶; Putnam *et al.*, 1999 ▶; Parikh, Putnam *et al.*, 2000 ▶; Handa *et al.*, 2001 ▶). Structural superposition also reveals that the ‘proline-rich loop’ (*XX*PPS) observed in other UNGs is missing in D4. How these varied motifs influence DNA binding in D4 is not known since no structure of any poxvirus UNG has been determined in the DNA-bound state. However, crystal structure analysis of free D4 revealed that five (Asp68, Tyr70, Phe79, Asn120 and His181) of the six residues defining the conserved uracil-recognition pocket in D4 are identical and the only different residue, Ile67, replaces the conserved glutamine. To further characterize the uracil-binding site in D4, we prepared the uracil complex of *Vaccinia virus* D4 by co-crystallization with uracil. Here, we report the crystal structure of the uracil complex of D4. In addition, we compare the interactions of uracil in the D4 active site with the observed interactions of uracil in complexes of *E. coli* UNG.

## 2. Materials and methods

### 2.1. Cloning, expression and purification

The coding sequence for D4 was engineered into the *Escherichia coli* expression vector pET15b and the recombinant protein was expressed in *E. coli* Rosetta (DE3) pLysS strain (EMD4Biosciences) by induction with 0.4 m*M* IPTG overnight at 295 K. The purification of recombinant protein has been described previously (Schormann *et al.*, 2007 ▶). Briefly, the protein was purified by metal-affinity chromatography using immobilized cobalt bound to TALON resin (Clontech Laboratories) and eluted in a gradient between ∼100 and 200 m*M* imidazole. After dialysis against 25 m*M* HEPES buffer pH 7.3, 300 m*M* KCl, 1 m*M* TCEP, the protein was concentrated to 9 mg ml^−1^. Frozen aliquots were stored at 193 K until use.

### 2.2. Crystallization, data collection and data processing

To prepare a complex of D4 with uracil we used a co-crystallization strategy. Prior to crystallization, we incubated 30 µl D4 (9 mg ml^−1^ or ∼330 µ*M* in 25 m*M* HEPES buffer pH 7.3, 0.3 *M* KCl, 1 m*M* TCEP) with 3 µl uracil (200 m*M* stock solution in 100% DMSO) for 1 h at 277 K. The mixture was used for crystallization by the hanging-drop vapor-diffusion method. 1 µl protein solution was mixed with an equal volume of reservoir solution. Rod-shaped and rectangular-shaped crystals with average dimensions of ∼0.18 × 0.12 mm were obtained after 1–2 d at 277 K. The reservoir solution of the crystal used for data collection consisted of 10% PEG 8000, 0.1 *M* Tris–HCl pH 8.0, 10% DMSO. To increase the DMSO concentration in the crystal for direct cooling, the cover slip containing the hanging drop was sealed over a new reservoir consisting of 10% PEG 8000, 0.1 *M* Tris buffer pH 8.0, 25% DMSO and the drop was then allowed to equilibrate for 3 d at 277 K. This crystal was flash-cooled directly in liquid nitrogen.

Data were collected on a Pilatus 6M detector at the Advanced Photon Source (beamline NE-CAT 24-ID-C) at 100 K. Previously, we noticed possible binding of DMSO near the active site of D4 in crystals that had been soaked in a solution containing DMSO. We therefore collected highly redundant X-ray data at a wavelength of 1.77 Å that should allow the verification of S atoms in an anomalous difference map. An initial data set of 120 oscillation images (1° per image) was collected at a crystal-to-detector distance of 150 mm. To increase the data multiplicity and improve the signal-to-noise ratio for the anomalous signal, two additional partially overlapping sweeps (200 images each, 1° per frame) were added. The three sweeps were then merged and scaled together. The highly redundant data (multiplicity of 17 overall and 10 in the highest resolution shell) were used to maximize the anomalous signal, which allowed the placement of different buffer components (potassium and chloride ions) and several DMSO molecules (five out of a total of ten) based on their anomalous scattering properties (anomalous scattering coefficients Δ*f*′′ at 1.77 Å: K^+^, 1.39e^−^; Cl^−^, 0.91e^−^; S, 0.74e^−^). The signal for S atoms in well ordered Cys and Met residues served as an internal control with regard to the quality of the anomalous difference map.

Data were processed with* XDS* (Kabsch, 2010*a* ▶,*b* ▶) and *SCALA* (Evans, 2006 ▶) in the *CCP*4 suite (Winn *et al.*, 2011 ▶) as part of the RAPD data-collection strategy at NE-CAT (https://rapd.nec.aps.anl.gov/rapd). Data-collection statistics are listed in Table 1 ▶.

### 2.3. Structure determination and refinement

The unit-cell parameters and the diffraction resolution suggested a value of 2.46 Å^3^ Da^−1^ for the Matthews coefficient and a solvent content of 50% for 12 subunits of D4 in the asymmetric unit. The crystal structure was solved by molecular replacement with *Phaser* (McCoy *et al.*, 2007 ▶) using the coordinates of one subunit from the D4 structure (PDB entry 4dof) as a search model. The σ_A_-weighted difference electron-density map (*mF*_o_ − *DF*_c_) at the 3σ contour level calculated after initial map fitting and refinement of the protein residues allowed the placement of uracil molecules in the catalytic pockets of all 12 subunits. The positions of the uracil in the active site were verified by the calculation of σ_A_-weighted difference maps (*mF*_o_ − *DF*_c_ ≥ 3σ) using *REFMAC* (Murshudov *et al.*, 2011 ▶) omitting the ligands from refinement and map calculation. Initially automatically generated NCS restraints were employed, and in later stages of the refinement we used loose NCS restraints. Prior to the final refinement cycles 1617 water molecules were added to the model at stereochemically appropriate locations in the difference electron-density map (*mF*_o_ − *DF*_c_ ≥ 3σ) using *Coot* (Emsley & Cowtan, 2004 ▶). *REFMAC* (v.5.7) was used for structure refinement and validation was performed using *MolProbity* (Chen *et al.*, 2010 ▶). Refinement statistics are listed in Table 1 ▶. The final atomic coordinates and structure factors for the uracil complex have been deposited in the PDB (entry 4lzb).

## 3. Results and discussion

### 3.1. Overall structure

**Figure 1:**
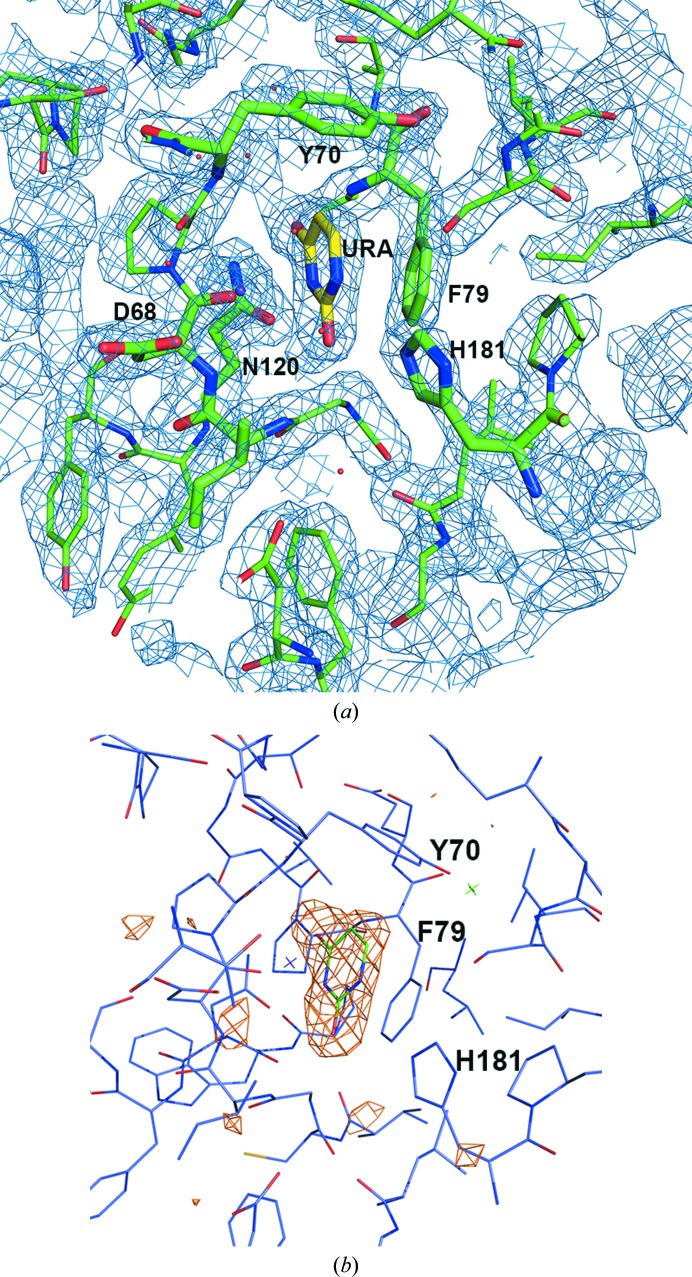
Electron-density maps. (*a*) Electron density representing the uracil molecule and nearby residues in the *A* subunit of the D4–uracil complex. Residues within 12 Å distance of the uracil base are shown as thin sticks and active-site residues are shown as thick sticks and labeled. The map shown is a σ_A_-weighted 2*mF*_o_ − *DF*_c_ map contoured at 1.5σ, where *m* is the figure of merit and *D* is the σ_A_ weighting factor. (*b*) Electron density representing the uracil molecule in the *A* subunit is shown. The electron-density map is an *mF*_o_ − *DF*_c_ map (*m* is the figure of merit and *D* is the σ_A_ weighting factor) calculated after the removal of uracil molecules from each subunit. The displayed map is contoured at 3σ and covers a distance of 8 Å around the uracil base.

The D4 protein used in this study contained an N-terminal hexahistidine tag and a thrombin cleavage sequence [Met-Gly-Ser-Ser-(His)_6_-Ser-Ser-Gly-Leu-Val-Pro-Arg-Gly-Ser-His]. The structure of the uracil complex was refined to 2.03 Å resolution (*R*_work_ = 21.2%, *R*_free_ = 24.6%). In the asymmetric unit five homodimers are formed between subunits related by noncrystallographic symmetry and two additional subunits form a dimer with their respective symmetry mates. The final model contains 2604 residues, of which 97.3% are in the favored region of the Ramachandran plot. A total of eight residues (Asp138 in seven subunits and Leu127 in one subunit) are in the disallowed region. The *MolProbity* (Chen *et al.*, 2010 ▶) clashscore of 1.23 (100th percentile for structures in the resolution range 2.03 Å) and overall score of 0.98 (100th percentile for all structures in the same resolution range) indicated that the quality of the model is excellent. Uracil molecules were successfully modeled in the active sites of all 12 subunits, consistent with a σ_A_-weighted 2*mF*_o_ − *DF*_c_ map at 1σ and a σ_A_-weighted *mF*_o_ − *DF*_c_ OMIT map at 3σ (see Supplementary Fig. S1^1^). Representative electron-density maps around the uracil-binding site in subunit *A* are shown in Fig. 1 ▶. The average *B* factor for uracil molecules (26.8 Å^2^) is similar to that for all protein residues (32.1 Å^2^). In addition, ten DMSO and 16 EDO molecules, ten potassium and 14 chloride ions, and 1617 water molecules were modeled. Occupancies for all atoms were kept at 1.0, except for the side-chain atoms of 36 residues which displayed alternate conformations; the occupancy for each atom in both conformations was fixed at 0.5. Potassium and chloride ions and five of the ten DMSO molecules were placed at the overlapping peak positions in an anomalous difference map contoured at ≥3σ (see Supplementary Fig. S2) and a σ_A_-weighted *mF*_o_ − *DF*_c_ electron-density map contoured at 3σ. Five additional DMSO molecules were placed based on the shape of the electron density. Peaks corresponding to the S atoms in the cysteine residues were observed at 4σ in the anomalous difference map. The potassium ions show coordination to O atoms (carbonyl, carboxylate, amide and hydroxyl groups and water molecules) at a distance of ∼2.3–3.4 Å. The observed distances are consistent with those reported previously for potassium ions in protein structures (MESPEUS database; Hsin *et al.*, 2008 ▶). The chloride ions show interactions with N atoms in lysine and arginine side chains and other atoms, including O atoms in water molecules. The interactions of potassium and chloride ions with protein residues, water and other ligands are listed in Supplementary Tables S1 and S2. Correlation coefficients and temperature factors of each ion are shown in Supplementary Table S3.

**Figure 2:**
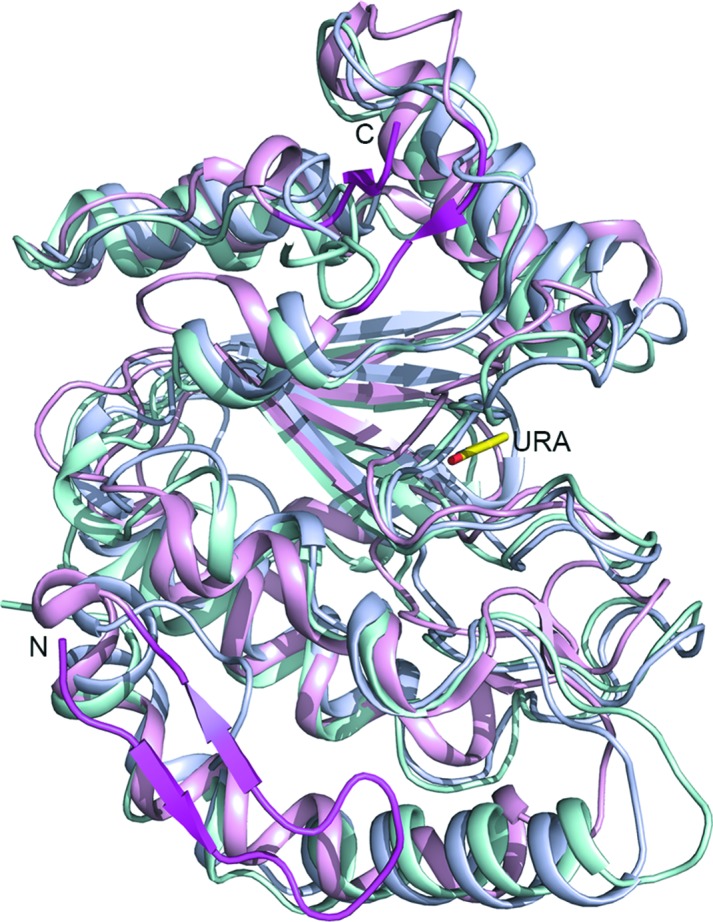
Structure of D4. Cartoon diagram showing a superposition of the structures of UNG from *E. coli* (bluish white) and human (light cyan) on D4 (light pink). The root-mean-squared deviations for superposition of the human and *E. coli* UNG structures on that of D4 are 4.51 and 3.76 Å, respectively. Distinctive structural elements of D4 are highlighted in light magenta. The uracil molecule in D4 is shown as a stick model.

In D4 there is a distinct two-stranded antiparallel β-sheet at the N-­terminus and additional short stretches of structural elements not seen in other UNG structures (Fig. 2 ▶). The structure of the D4 molecules in the complex is nearly identical to the structure of the free enzyme described previously (Schormann *et al.*, 2007 ▶). The three-dimensional structure of D4 exhibits the core elements of UNG structures: a four-stranded β-sheet sandwiched between four α-­helices on both sides. The conformational differences between the 12 subunits are small (the r.m.s.d. of 213 aligned C^α^ atoms is 0.43 Å). The largest deviations between individual subunits are observed near the leucine-intercalation loop (residues 183–189) and in the loop/helix region (residues 164–173). The same areas also showed high flexibility in uracil-free D4 (PDB entry 2owr; see Supplementary Fig. S3).

### 3.2. Uracil binding in D4

**Figure 3:**
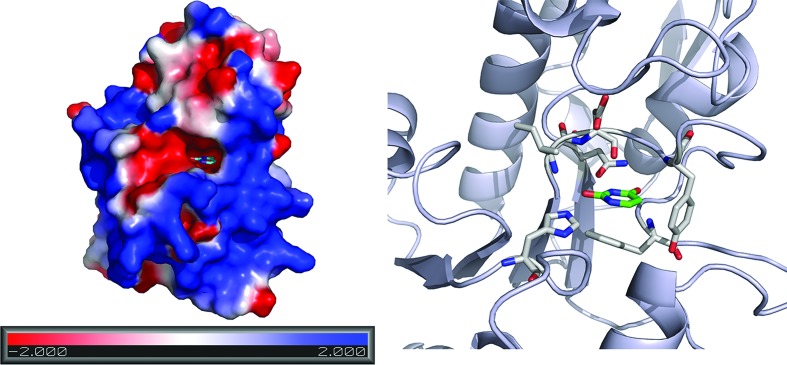
Uracil-binding pocket. Surface diagram (left) and cartoon diagram (right) showing the uracil-binding pocket in D4. Electrostatic potential was calculated using *DelPhi* (Rocchia *et al.*, 2001 ▶) and plotted using the *APBS* plugin in *PyMOL* (DeLano, 2002 ▶). The uracil molecule and active-site residues are shown as stick models.

As shown in Fig. 3 ▶, the uracil molecule binds deep inside the pocket formed by Ile67, Asp68, Tyr70, Phe79, Asn120 and His181. The orientation of the uracil molecule and its interactions with the protein are consistent with those observed in other UNGs [eUNG (PDB entries 2eug and 1flz; Xiao *et al.*, 1999 ▶; Werner *et al.*, 2000 ▶), hsvUNG (PDB entry 1udh; Savva *et al.*, 1995 ▶) and hUNG (PDB entries 1ssp and 1emj; Parikh *et al.*, 1998 ▶; Parikh, Walcher *et al.*, 2000 ▶)]. The uracil ring in D4 is positioned nearly perpendicular to the aromatic ring of Tyr70, with the C5 atom of uracil at a distance of less than 4.0 Å from all atoms of the tyrosine ring. A similar packing of uracil has been noticed in other UNGs and the close proximity of the tyrosine to the C5 atom of uracil and the specific aromatic environment of the uracil base have been shown to be important for the uracil specificity of UNGs (Savva *et al.*, 1995 ▶; Parikh *et al.*, 1998 ▶; Xiao *et al.*, 1999 ▶). The OD1 and ND2 atoms of the side chain of Asn120 show hydrogen bonds to the N3 and O4 atoms of uracil. The O2 and O4 atoms of uracil form hydrogen bonds to the peptide N atoms of Ile67 and Phe79. As noticed in *E. coli* and human UNG, a conserved water molecule is observed at a distance of 2.9–3.2 Å from the O4 atom of uracil (Table 2 ▶).

**Figure 4:**
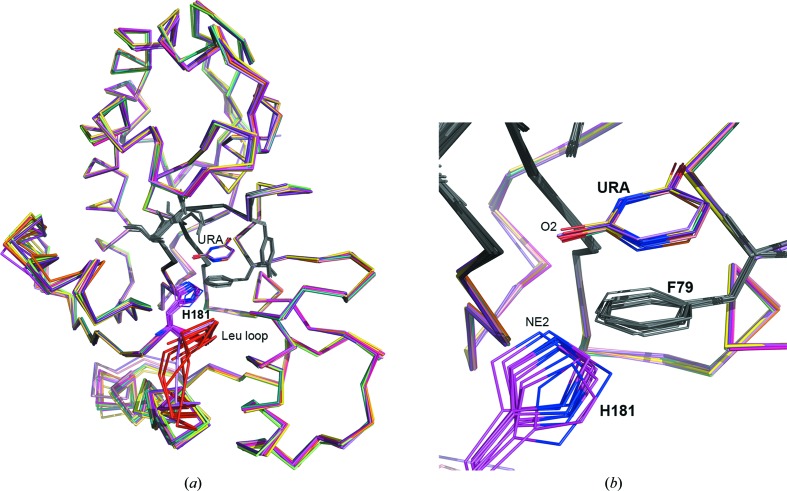
Movement of the His181 side chain. (*a*) Cartoon diagram showing superposition of various subunits of the uracil complex. The uracil and active-site residues are shown as lines (gray, except for His181, which is colored by atom: C, magenta; N, blue; O, red). The region of the leucine-intercalation loop is colored red (labeled ‘Leu loop’). (*b*) Close-up view of His181 and uracil (both labeled) in the active site of various subunits.

In the present structure, the amino-acid residues in the active sites (except His181) of all subunits superpose well. The r.m.s.d. for pairwise alignment of the C^α^ atoms of all residues in various subunits within 8 Å of the uracil molecule is between 0.14 and 0.22 Å. However, the position of the side chain of His181 varies in different subunits. As a result of this movement, the distance between the NE2 atom of His181 and the O2 atom of uracil varies from 2.8 to 4.8 Å in different subunits (Table 2 ▶; Fig. 4 ▶). Previously, uracil has been located in UNG structures as a reaction product (eUNG, PDB entry 1flz; hUNG, PDB entries 1ssp and 1emj), as part of uncleaved pseudo­uridine-containing DNA (hUNG, PDB entry 1emh) and as a free ligand (eUNG, PDB entry 2eug; hsvUNG, PDB entry 1udh). In UNGs the active-site histidine, which serves as a neutral electrophile, donates a hydrogen bond to the O2 atom of uracil in the transition state. The distance between the active-site histidine and uracil is significantly shorter in productive complexes with UNGs. For example, when uracil was soaked into crystals of eUNG and hsvUNG the observed distances between these two atoms were 4.2 Å (PDB entry 2eug) and 5.1 Å (PDB entry 1udh), respectively (Xiao *et al.*, 1999 ▶; Savva *et al.*, 1995 ▶). On the other hand, when uracil was captured as a reaction product in eUNG the corresponding distance was 3.0 Å (Werner *et al.*, 2000 ▶). Similarly, in hUNG–product complexes (PDB entries 1ssp and 1emj) the NE2 atom of histidine is at a distance of 2.7 and 2.9 Å from the uracil O2 atom (Parikh *et al.*, 1998 ▶; Parikh, Walcher *et al.*, 2000 ▶). In the D4–uracil complex the distance between His181 NE2 and uracil O2 is less than 3.0 Å in four subunits (*C*, *G*, *I* and *K*), while in four other subunits (*A*, *B*, *F* and *H*) the observed distance is greater than 3.5 Å. In the remaining four subunits the distance ranges between 3.0 and 3.5 Å (see Table 2 ▶). However, the uracil in this complex does not represent an active product and the His181 residue lies in a highly flexible area of the molecule; therefore, the movement of its side chain may not be related to ligand binding.

**Figure 5:**
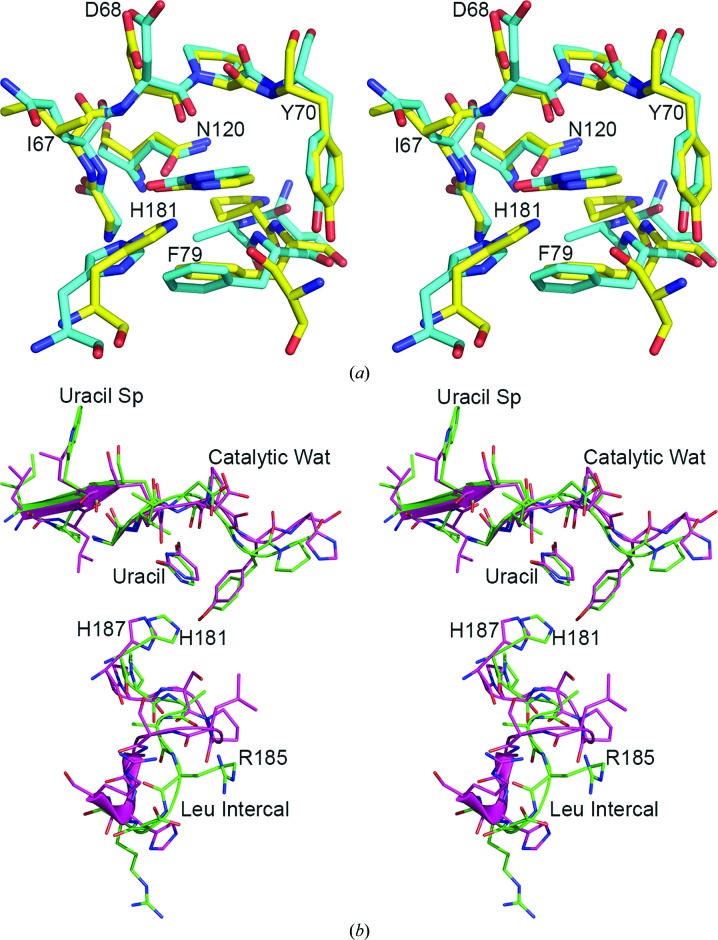
Comparison of *E. coli* UNG and D4. (*a*) Stereo diagram showing a superposition of amino-acid residues within 4.5 Å distance of uracil in *E. coli* UNG (PDB entry 2eug) and D4. Amino-acid residues are shown as stick models. Color code for D4: N, blue; O, red; C, yellow; *E. coli* UNG is shown in cyan. (*b*) Stereo drawing showing superposition of the uracil-specificity motif, catalytic water loop and leucine-intercalation loop in *E. coli* UNG (PDB entry 2eug) and the corresponding regions in D4 (subunit *B*). The active-site histidines His187 of *E. coli* UNG and His181 of D4 are labeled. Color code for D4: N, blue; O, red; C, green in D4; *E. coli* UNG is shown in magenta .

Superposition of the residues within 4.5 Å of the uracil molecule in the nonproductive uracil complex of eUNG (PDB entry 2eug) and D4 shows that despite some differences in the composition of the uracil-binding pocket, the amino-acid residues lining the pocket align well (Fig. 5 ▶ *a*). Primary-sequence alignment and three-dimensional structural superposition of D4 and other UNGs reveal significant differences in the amino-acid sequences in regions (of D4) corresponding to various conserved UNG-specific motifs. Superposition of the residues representing the uracil-specificity motif, catalytic water loop and leucine-intercalation loop in eUNG with corresponding residues in D4 showed a significant divergence in the structure of the leucine-intercalation loop (Fig. 5 ▶ *b*). The location of the uracil molecule is remarkably similar.

**Figure 6:**
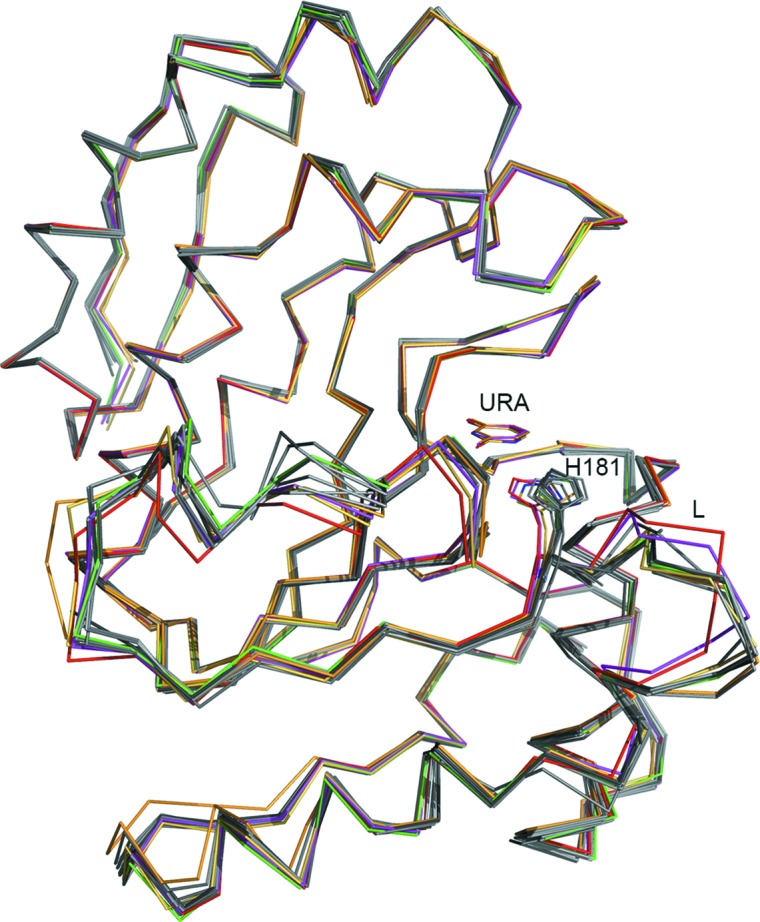
Ribbon diagram of free D4 and the uracil complex, showing superposition of all eight subunits of the uracil-free D4 structure (PDB entry 2owr; gray to black) and four subunits of the uracil complex (*B*, *C*, *D* and *G*) in red, orange, magenta and green. Uracil molecules and His181 are shown as line drawings. The leucine-intercalation loop is labeled L.

Since crystal structures of D4 both in the free state (PDB entry 2owr) and in the uracil-bound state contain multiple copies in the asymmetric unit (eight and 12 subunits, respectively), these structures allow us to visualize the conformational flexibility in different areas of the protein molecule. Compared with the uracil-free D4 structure, conformational differences in the D4 subunits of the complex are restricted mainly to the leucine-intercalation loop and the loop–helix–loop stretch (residues 161–175; Fig. 6 ▶). The positions of all active-site residues including the catalytic aspartic acid (Asp68) remain unchanged. Biochemical, mutational and structural studies on a number of UNGs have established that the conserved leucine residue (of the leucine-intercalation loop) plays a crucial role in nucleotide flipping and catalysis (Savva & Pearl, 1995 ▶; Savva *et al.*, 1995 ▶; Parikh *et al.*, 1998 ▶; Handa *et al.*, 2001 ▶; Jiang & Stivers, 2002 ▶). Based on structural superimposition, the corresponding residue in D4 is Arg185. Yet, how the conformational shift in the intercalation loop that results from DNA binding repositions Arg185 remains to be elucidated. Nevertheless, the structure of the complex presented here reveals that despite the differences in the characteristic motifs, the architecture of the uracil-binding pocket and the interactions of the amino-acid residues with uracil are conserved in D4.

## Supplementary Material

PDB reference: uracil DNA glycosylase, complex with uracil, 4lzb

Supplementary material file. DOI: 10.1107/S1744309113030613/en5544sup1.pdf

## Figures and Tables

**Table 1:** Data-collection and refinement summary Values in parentheses are for the highest resolution shell.

Data-collection statistics	
Wavelength (Å)	1.77
Space group	*P*2_1_2_1_2_1_
Unit-cell parameters (Å)	*a* = 93.47, *b* = 114.05, *c* = 302.52
Resolution range (Å)	49.64–2.03 (2.14–2.03)
Total No. of observations	3383622 (271866)
No. of unique reflections	198586 (26784)
Multiplicity	17.0 (10.2)
Completeness (%)	95.2 (88.9)
*R*_merge_ †	0.130 (0.584)
*R*_meas_(*I*)‡	0.142 (0.628)
*R*_p.i.m._(*I*)‡	0.034 (0.171)
〈*I*/σ(*I*)〉	25.9 (3.9)
Refinement statistics	
Resolution range (Å)	49.64–2.03 (2.08–2.03)
No. of unique reflections	198460 (13278)
Completeness (%)	94.9 (86.8)
*R*_cryst_ § (%)	21.3 (26.6)
*R*_free_ § (%)	24.6 (30.5)
No. of protein atoms	21390
No. of heteroatoms	224
No. of water molecules	1617
Wilson *B* factor (Å^2^)	27.8
Average *B* factors (Å^2^)	
Overall	31.9
Protein atoms	32.1
Water molecules	35.4
Ligand (uracil)	26.8
Potassium ions	37.1
Chloride ions	33.7
Coordinate error (ESU)	0.13
Correlation coefficient, *F*_o_ − *F*_c_	0.93
Correlation coefficient, *F*_o_ − *F*_c,free_	0.92
Ramachandran plot, residues in (%)	
Allowed region	99.7
Disallowed region	0.30
*MolProbity* scores	
Clashscore	1.23 [100th percentile]
Overall score	0.98 [100th percentile]

† *R*_merge_ = 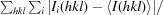 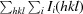.

‡ *R*_meas_ and *R*_p.i.m._ were calculated with *SCALA* (Evans, 2006 ▶) in the *CCP*4 suite (Winn *et al.*, 2011 ▶) using unmerged and unscaled data pre-processed by *XDS* (Kabsch, 2010*a* ▶,*b* ▶). *R*_meas_ is a merging *R* factor independent of data redundancy, while *R*_p.i.m._ provides the precision of the averaged measurement, which improves with higher multiplicity (Weiss, 2001 ▶). *R*_meas_ = 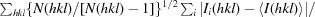 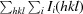. *R*_p.i.m._ = 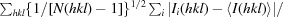 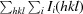

§ The data included in the *R*_free_ set (5%) were excluded from refinement.

**Table 2:** Uracil interactions (hydrogen-bonding distances in Å)

ID	O4–Wat	O4–Phe79 NH2	O4–Asn120 ND	O2–Ile67 NH	O2–His181 NE2	N3–Asn120 OD1	N3–Asp68 O
*A*308	2.86	2.73	2.86	3.19	3.52	2.68	2.92
*B*312	2.91	2.74	2.97	3.21	4.82	2.81	2.81
*C*303	2.85	2.86	2.85	3.05	2.97	2.75	2.86
*D*305	2.97	2.82	2.81	3.15	3.26	2.74	2.79
*E*307	2.86	2.83	2.94	3.20	3.24	2.79	2.77
*F*305	2.92	2.87	2.93	3.23	4.28	2.81	2.88
*G*306	2.88	2.78	2.85	3.12	2.99	2.74	2.85
*H*304	3.20	2.82	2.95	3.11	3.71	2.80	2.84
*I*303	2.92	2.68	3.01	2.98	2.84	2.86	2.73
*J*301	2.92	2.88	2.76	3.06	3.06	2.73	2.77
*K*303	2.92	2.75	2.94	3.08	2.97	2.85	2.81
*L*302	3.07	2.70	2.97	3.08	3.43	2.72	2.79

## Footnotes

1 Supplementary material has been deposited in the IUCr electronic archive (Reference: EN5544).
