# Supplementary material for: Structure of the uracil complex of Vaccinia virus uracil DNA glycosylase
Source: Acta Crystallogr Sect F Struct Biol Cryst Commun. 2013 Nov 28;69(Pt 12):1328–34. doi: 10.1107/S1744309113030613 (PMC3855713; doi:10.1107/S1744309113030613)
Supplement: Supplementary file 1 [file f-69-01328-sup1.pdf]

Supplementary Material

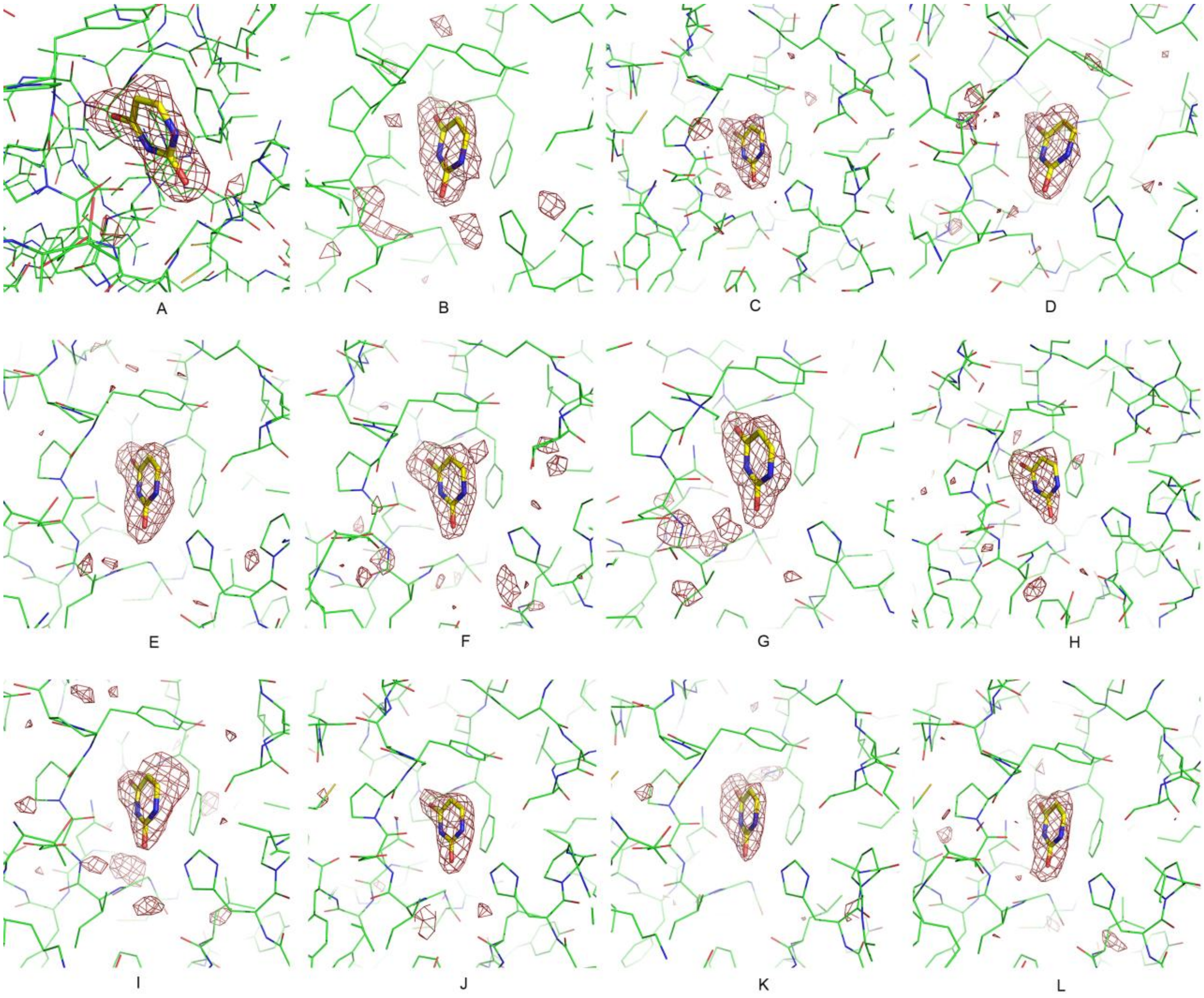

Supplementary Figure S1. Electron density representing the uracil molecule in each subunit is shown. The electron density map is a mFo-dFc map (‘m’ is the figure of merit and ‘d’ is the SigmaA weighting factor) calculated after removal of uracil molecules from each subunit. The displayed maps are contoured at 3σ and cover 8 Å distance around the uracil base.

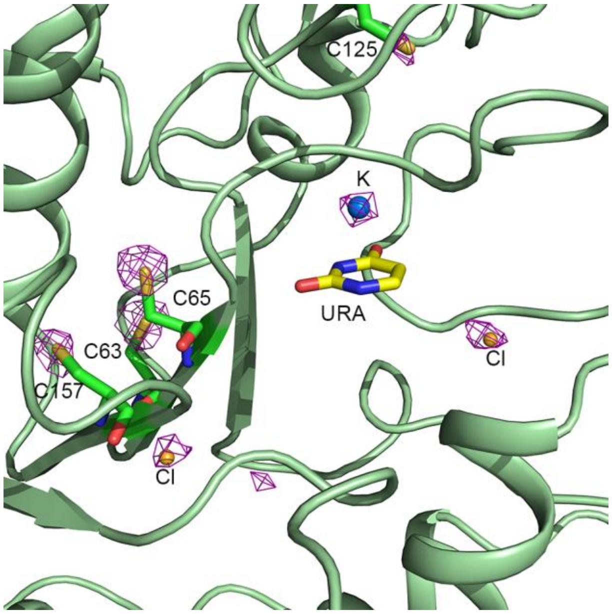

Supplementary Figure S2. Anomalous difference electron density map (red mesh) contoured at 4σ covering 10 Å around uracil (shown in stick model). Peak positions representing location of SG of C65, potassium and chloride ions are shown.

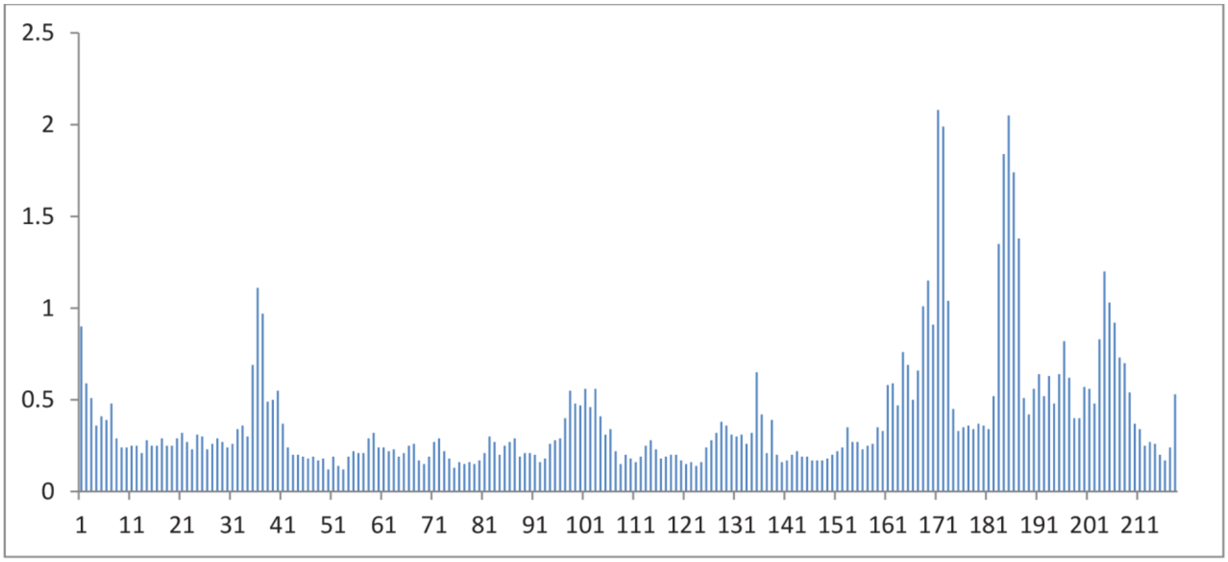

Uracil complex

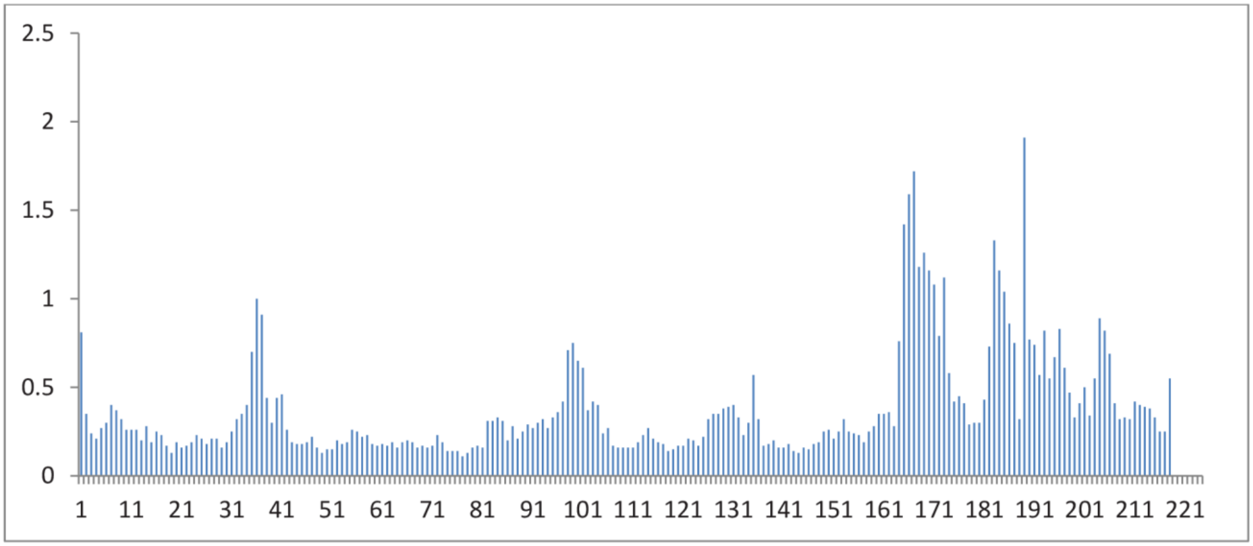

2OWR

Supplementary Figure S3. Root mean squared deviation (in Å, Y-axis) in the Cα positions of all residues in the 8 subunits of the uracil-free D4 structure (2OWR) and 12 subunits of the uracil complex are plotted against residue numbers (X-axis).

Supplementary Table S1. Potassium interactions (distances in Å).

ID†	P69_O	D68_O	D68_OD1	Water	Water	Water
A302	3.03	2.98	2.68	2.65	2.68	2.82
B305	2.87	3.01	2.72	2.81	3.33	
C301	3.00	3.01	2.68	2.55	2.63	
D301	3.12	2.98	2.77	2.75		
E303	3.01	3.07	2.57	2.60	2.76	2.84
F302	2.99	2.97	2.77	2.95	3.36	
G303	3.04	3.14	2.67	2.44	3.02	3.32
J303	3.13	3.30	2.79	2.53	2.69	2.70
	T149_O	V152_O	P173_O	EDO_O	Water	Water
B304	2.67	2.63	2.81	2.99	2.65	2.84
E302	2.61	2.65	2.71		2.33	2.89

† The first letter denotes the subunit (or chain) and the number represents the ion.

Supplementary Table S2. Chloride interactions (distances in Å)

ID[†]	H181_NH	K160_NH	Water	Water
A303	3.24	3.29	3.30	
B307	3.25	3.27	3.13	3.61
C302	3.15	3.27		
D302	3.20	3.23	4.05	
E304	3.21	3.45		
F303	3.32	3.17	3.07	
G305	3.17	3.39	3.85	
H301	3.11	3.39		
K301	3.22	3.22	4.21	

	K87_NH	S88_NH	S88_OG	Water	Water	Water
A304	3.81	3.19	2.91	3.16	3.71	4.08
E305	3.95	3.29	3.07	2.90	3.73	4.00
G301	3.86	3.25	3.23	2.94	3.74	4.04

	R167_NE	T176_NH	Water	Water
B306*	3.33/3.56	3.31/3.40	3.07	3.20

	A208_NH	R61_NE	Water
B308	3.00	4.26	3.02

[†] The first letter denotes the subunit (or chain) and the number represents the ion.

*indicates an ion at the dimer interface of subunits B and E.

Supplementary Table S3. Correlation coefficient and B-factor of K and Cl ions

Residue number / Correlation coefficient (map calculated from mtz file versus map calculated from coordinates) / B factors

ID†	Corr-coeff (K)	B (K)
A302	0.9211	42.0
B304	0.8958	16.8
B305	0.9192	38.9
C301	0.9635	42.7
D301	0.9287	51.2
E302	0.9031	19.1
E303	0.9596	40.7
F302	0.9524	41.5
G303	0.9481	34.1
J303	0.9624	43.7
Average (low, high)	0.94 (0.90, 0.96)	37.1 (16.8, 51.2)

ID†	Corr-coeff (CL)	B (CL)
A303	0.9475	30.5
A304	0.9266	36.8
B306	0.8767	15.9
B307	0.9452	25.5
B308	0.9398	36.6
C302	0.9318	39.6
D302	0.9152	39.2
E304	0.9519	29.3
E305	0.8975	38.3
F303	0.9360	26.5
G301	0.9262	36.7
G305	0.9396	37.5
H301	0.9265	39.5
K301	0.9462	39.8
Average (low, high)	0.93 (0.88, 0.95)	33.7 (15.9, 39.8)

† The first letter denotes the subunit (or chain) and the number represents the ion.
